# Surface Spectroscopy on UHV-Grown and Technological Ni–ZrO_2_ Reforming Catalysts: From UHV to Operando Conditions

**DOI:** 10.1007/s11244-016-0678-8

**Published:** 2016-08-12

**Authors:** Kresimir Anic, Astrid Wolfbeisser, Hao Li, Christoph Rameshan, Karin Föttinger, Johannes Bernardi, Günther Rupprechter

**Affiliations:** 1 Institute of Materials Chemistry, Technische Universität Wien, Getreidemarkt 9/BC/01, 1060 Vienna, Austria; 2 University Service Center for Transmission Electron Microscopy, Technische Universität Wien, Wiedner Hauptstraße 8-10, 1040 Vienna, Austria

**Keywords:** Nickel, Zirconia, Model catalysts, Technological catalysts, Carbon monoxide, Methane steam reforming, Methane dry reforming, In situ spectroscopy, Operando spectroscopy

## Abstract

Ni nanoparticles supported on ZrO_2_ are a prototypical system for reforming catalysis converting methane to synthesis gas. Herein, we examine this catalyst on a fundamental level using a 2-fold approach employing industrial-grade catalysts as well as surface science based model catalysts. In both cases we examine the atomic (HRTEM/XRD/LEED) and electronic (XPS) structure, as well as the adsorption properties (FTIR/PM-IRAS), with emphasis on in situ/operando studies under atmospheric pressure conditions. For technological Ni–ZrO_2_ the rather large Ni nanoparticles (about 20 nm diameter) were evenly distributed over the monoclinic zirconia support. In situ FTIR spectroscopy and ex situ XRD revealed that even upon H_2_ exposure at 673 K no full reduction of the nickel surface was achieved. CO adsorbed reversibly on metallic and oxidic Ni sites but no CO dissociation was observed at room temperature, most likely because the Ni particle edges/steps comprised Ni oxide. CO desorption temperatures were in line with single crystal data, due to the large size of the nanoparticles. During methane dry reforming at 873 K carbon species were deposited on the Ni surface within the first 3 h but the CH_4_ and CO_2_ conversion hardly changed even during 24 h. Post reaction TEM and TPO suggest the formation of graphitic and whisker-type carbon that do not significantly block the Ni surface but rather physically block the tube reactor. Reverse water gas shift decreased the H_2_/CO ratio. Operando studies of methane steam reforming, simultaneously recording FTIR and MS data, detected activated CH_4_ (CH_3_ and CH_2_), activated water (OH), as well as different bidentate (bi)carbonate species, with the latter being involved in the water gas shift side reaction. Surface science Ni–ZrO_2_ model catalysts were prepared by first growing an ultrathin “trilayer” (O–Zr–O) ZrO_2_ support on an Pd_3_Zr alloy substrate, and subsequently depositing Ni, with the process being monitored by XPS and LEED. Apart from the trilayer oxide, there is a small fraction of ZrO_2_ clusters with more bulk-like properties. When CO was adsorbed on the (fully metallic) Ni particles at pressures up to 100 mbar, both PM-IRAS and XPS indicated CO dissociation around room temperature and blocking of the Ni surface by carbon (note that on the partially oxidized technological Ni particles, CO dissociation was absent). The Ni nanoparticles were stable up to 550 K but annealing to higher temperatures induced Ni migration through the ultrathin ZrO_2_ support into the Pd_3_Zr alloy. Both approaches have their benefits and limitations but enable us to address specific questions on a molecular level.

## Introduction

Ni–ZrO_2_ catalysts are among the most important industrial catalysts, due to their widespread application for methane reforming reactions. Steam reforming [1–3], dry reforming [4–9] and partial oxidation [10, 11] are routes to produce synthesis gas (CO and H_2_) of varying composition but in all cases deactivation by coke (carbon filament) formation is a major limitation of catalyst performance and lifetime. Several strategies were applied to reduce coking, including alloying with a second metal (e.g. Au [12–14], Cu [15–25], intended to decorate the active Ni steps that are prone to coking with a less active metal) and/or using mixed-oxide supports [4, 5, 26–33]. Adding CeO_2_ to ZrO_2_ to has been found to improve the oxygen storage capacity and redox properties, which affects the ability to deliver oxygen from the lattice to carbon, increasing thermal stability and catalytic activity [3, 5, 26].

Apart from these modified variants, the “simple” Ni–ZrO_2_ catalysts still holds potential for fundamental studies of the nanoparticle size, shape and surface structure, of the interaction of the Ni nanoparticles with the oxide support, of the specific adsorption and reaction properties of metal-, oxide- and metal-oxide-interface sites, and of the thermal and chemical stability of the catalysts. Due to the relatively harsh reforming conditions, especially high temperature, the exact reaction mechanisms on Ni (and Pt) are still debated [34–39]. Herein, we describe an approach to examine fundamental properties of Ni–ZrO_2_ by performing surface spectroscopic studies both on technological and model (ultrahigh vacuum (UHV) prepared) catalysts. Whereas the first provide a link to industrial catalysis, the latter enable us to reduce complexity and to apply surface science techniques [40–51].

It is important to note that in both cases structure, composition and adsorption properties were examined which clearly reveals analogies but also differences between applied and model catalysts. For both types of catalysts we have performed “in situ” studies, i.e. acquiring surface spectra during ongoing processes, in an effort to obtain meaningful and technologically relevant structure–activity correlations. In recent years we have applied this approach to a variety of technological catalysts [52–58] and single-crystal based model catalysts [59–61]. For the technological catalysts, when catalytic activity is recorded simultaneously, the term “*operando*” spectroscopy is rather used [62, 63].

## Experimental

In the following, the synthesis/preparation of the Ni–ZrO_2_ catalysts is briefly described. For more detailed descriptions we refer to refs [24, 25].

### Impregnated Ni–ZrO_2_

The technological Ni–ZrO_2_ catalyst was prepared by impregnation of ZrO_2_ (commercial Zr(OH)_4_ from MEL chemicals XZO 880/01; calcined at 973 K) with a total metal weight content of 5 % w/w. After dissolving the appropriate amount of Ni-nitrate in water, ZrO_2_ powder was suspended in the solution, which was dried at 373 K overnight. Then the powder was heated to 723 K in air with a heating rate of 5 K min^−1^ and calcined at 723 K for 2 h. Prior to characterization and reaction the catalyst was oxidized at 773 K and reduced at 673 K (at ~1 bar in 20 % O_2_ in Ar and 20 % H_2_ in Ar, respectively, with a flow rate 50 ml min^−1^).

The catalyst was characterized by high resolution transmission electron microscopy (HRTEM) including energy dispersive X-ray fluorescence (EDX) analysis, H_2_-chemisorption, infrared spectroscopy (FTIR) with CO as a probe molecule, X-ray diffraction (XRD), and X-ray absorption spectroscopy (XAS). To study the catalytic performance, the catalyst was tested in a continuous flow setup for methane dry and steam reforming. Operando spectroscopy provides information about the catalyst under reaction conditions, i.e. high temperature and reactive gas atmosphere. In this respect, operando IR, XAS and near atmospheric pressure X-ray photoelectron (NAP-XPS) spectroscopy were applied. After reaction, the used catalyst was further studied via temperature programmed oxidation (TPO), in order to get information about the amount of carbon deposition and the temperature needed to remove the carbon species via oxidation, and by HRTEM, imaging carbon deposition.

### Ni Nanoparticles on Ultrathin ZrO_2_ Films Grown in UHV

The experimental setup used to prepare and characterize the ultrathin ZrO_2_ support, as well as the Ni nanoparticles, has been described in detail in references [49, 50]. In brief, it consists of an ultrahigh vacuum (UHV) chamber for sample preparation and characterization (LEED, TPD, XPS, IRAS), which is connected to a UHV-compatible atmospheric pressure reaction cell (“Rupprechter-design” [64]) that also allows for in situ PM-IRAS spectroscopy. In order to prepare an ultrathin model zirconia thin film with Ni nanoparticles on top, either a Pd_3_Zr(0001) or Pt_3_Zr(0001) single crystal was cleaned by sputtering (8 × 10^−6^ mbar of Ar, 2 kV) and annealing in UHV. After cleaning, the Pd_3_Zr or Pt_3_Zr single crystal was exposed to 1 × 10^−7^ mbar of O_2_ at 673 K for 30 min, followed by annealing in UHV at 1073 K for 20 min to grow an uniform ultrathin ZrO_2_ layer (for details see [65, 66]). On top of this ultrathin layer, an amount of Ni equivalent to a homogeneous layer of nominally 3 Å was deposited at room temperature, utilizing an Omicron electron-beam triple evaporator (EFM 3T) for physical vapor deposition. The prepared model catalyst surface was characterized by LEED and XPS and, to follow the interaction of the surface with CO, by polarization modulation infrared reflection absorption spectroscopy (PM-IRAS) [67].

## Results and Discussion

The following section is divided into two parts, the first describing results obtained for the technological catalyst, and the second describing those for the model catalyst. For both we first focus on structural properties, then turn to CO adsorption properties, and finally discuss studies performed under elevated pressure and/or elevated temperature conditions.

### Technological Ni–ZrO_2_

#### Structure Characterization

Applying H_2_-chemisorption to Ni–ZrO_2_ after oxidation at 773 K and reduction at 673 K indicated an accessible metallic Ni surface area of 31.4 m^2^ g^−1^ [68]. This results in a calculated Ni particle size of 21.5 nm (implying spherical Ni particles) which is in reasonable agreement with the particle size found by TEM, as described below. Note however, that for XRD and TEM analysis, the reduced Ni–ZrO_2_ had to be exposed to air prior to the measurements. Due to easy reoxidation, at least the outer shell of the Ni nanoparticles is thus not expected to remain in the metallic state.

**Fig. 1:**
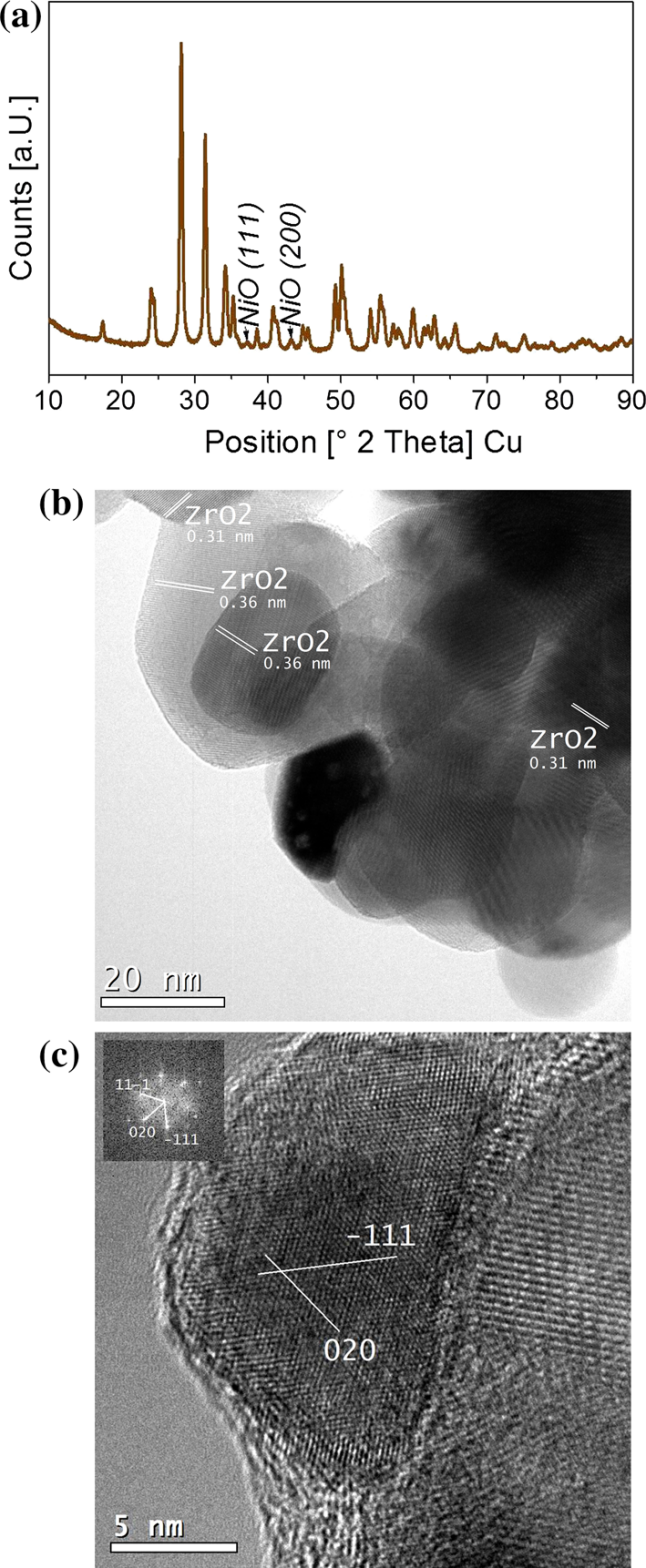
XRD (**a**) und TEM (**b**, **c**) of technological Ni–ZrO_2_ after ex situ oxidation at 773 K followed by reduction at 673 K. The sample was exposed to air prior to XRD and TEM, however. The XRD pattern shows reflections characteristic of monoclinic ZrO_2_ and cubic NiO. TEM reveals ZrO_2_ (**b**) and Ni/NiO particles (**c**) of about 50 and 20 nm diameter, respectively

The diffractogram in Fig. 1a) displays mostly reflections characteristic of monoclinic ZrO_2_. Small peaks of cubic NiO (at 37.3° and 43.2°) are visible, whereas those of metallic Ni (at 44.4° and 51.8° and also expected to be weak) are overlapping with ZrO_2_ reflections. According to TEM, the particles of the monoclinic ZrO_2_ support have a diameter of about 50–100 nm (Fig. 1b). At low magnification the low Z-contrast between zirconia and Ni or NiO prevents the unambiguous identification of Ni/NiO particles and EDX was thus applied to locate them. Typically, the particles consisted of a Ni core and a disordered shell (with a thickness of up to several nanometers). The fcc Ni particle core is imaged with lattice resolution in Fig. 1b. According to XRD, the shell must be NiO. The Ni/NiO particles were evenly distributed on the zirconia support and their size ranged from about 10–30 nm, but most of them had a diameter of about 20 nm.

#### CO Adsorption

**Fig. 2:**
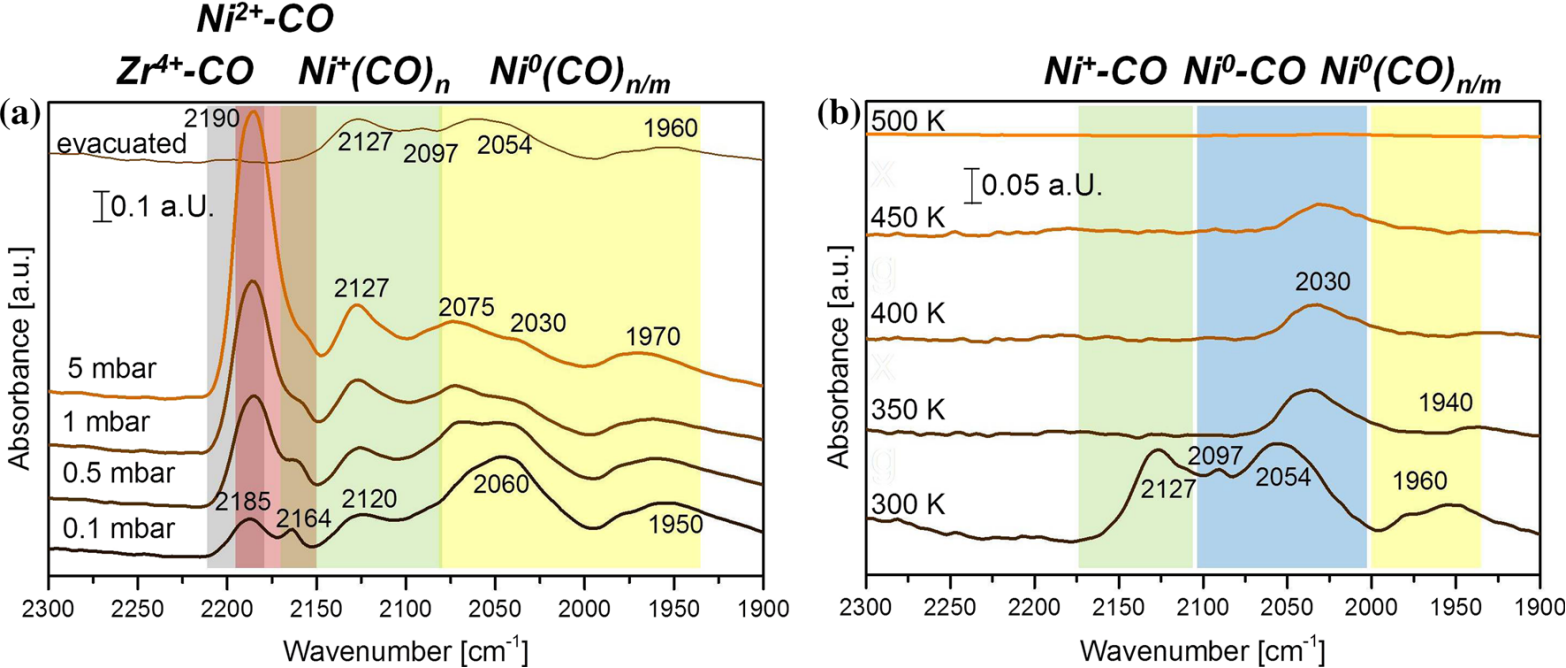
**a** FTIR spectra of Ni–ZrO_2_ (reduced in 5 mbar H_2_ at 673 K) acquired at 300 K during CO adsorption at different pressures and after evacuation. **b** The thermal stability of adsorbed CO was followed by recording FTIR spectra upon annealing in vacuum (heating rate of 10 K min^−1^ *)*

In the next step, the interaction of the technological catalyst with CO was studied by FTIR. For the adsorption experiments Ni–ZrO_2_ was pre-reduced in 5 mbar H_2_ at 673 K. Afterwards 0.1, 0.5, 1.0, and 5.0 mbar CO were dosed at 300 K and for each respective pressure FTIR spectra were acquired (Fig. 2a). Additionally, one spectrum was recorded after evacuation to less than 2 × 10^−6^ mbar.

The peaks at 2185–2190 and 2164 cm^−1^ are attributed to Zr^4+^–CO and Ni^2+^–CO, respectively. With increasing CO pressure, the Zr^4+^–CO peak grows and obscures the Ni^2+^–CO peak. The band at 2060 cm^−1^ appearing at 0.1 mbar pressure is attributed to linearly adsorbed CO on Ni^0^ and the broad band(s) below 2000 cm^−1^ are attributed to threefold hollow bonded CO on Ni^0^ [69–71]. The band at 2120 cm^−1^, shifting to 2127 cm^−1^ with increasing CO pressure, is attributed to Ni^+^–CO. The unusual oxidation state of Ni^+^ is stabilized by the ligand CO [72]. Indeed, the decreasing peaks of CO on Ni^0^ and Ni^2+^ and the increasing CO–Ni^+^ peak are due to a surface reaction in the presence of CO (Ni^2+^ + Ni^0^ → 2 Ni^+^; see Kasal et al. [72]).

After evacuation, Ni^+^–CO as well as linear and hollow bonded CO on Ni^0^ partly remain on the surface. Overall, CO FTIR indicates that, in agreement with XAS measurements [68], both reduced and oxidized Ni species are present on the catalyst surface and that NiO reduction in H_2_ is not complete at 673 K.

This may explain why CO dissociation (and resulting carbon poisoning) is not occurring for the technological catalyst upon room temperature CO adsorption. When the CO dosing was repeated (after evacuation) basically the same IR spectra were obtained. As shown below, the supported (fully metallic) Ni nanoparticles prepared in UHV dissociated CO already around room temperature.

In a consecutive experiment (after evacuation) CO desorption was monitored via heating to elevated temperatures. In Fig. 2b the thermal stability of adsorbed CO was followed by recording FTIR spectra during heating with a rate of 10 K min^−1^ in high vacuum. CO desorbed from Ni^+^ between 300 and 350 K, adsorbed multiply coordinated CO on Ni^0^ vanished at 400–450 K (in agreement with single crystal data [64, 73]) and at 450–500 K all CO (including linear) had desorbed from Ni–ZrO_2_.

#### Methane Dry Reforming (MDR)

The most straightforward and direct measurement of the thermal stability of the Ni–ZrO_2_ catalyst is via its catalytic activity at high temperature, such as during methane dry reforming:

$$CH_{4} + CO_{2} \,\leftrightarrows\, 2 CO + 2 H_{2} \quad \Delta H_{298 K}^{^\circ } = + 261 kJ/mol.$$

**Fig. 3:**
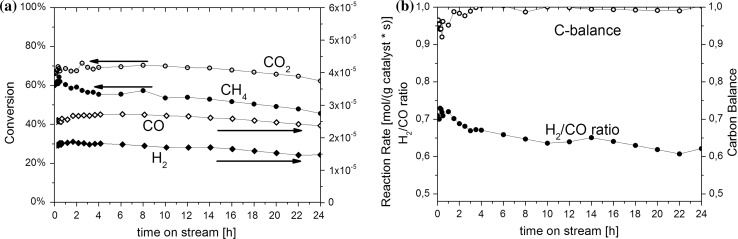
**a** Conversion of reactants and reaction rate of hydrogen and carbon monoxide production and **b** H_2_/CO ratio and carbon balance over Ni–ZrO_2_ for methane dry reforming reaction at 873 K; feed composition: CH_4_:CO_2_:Ar = 10/10/80; feed flow rate = 25 ml min^−1^

After oxidation at 773 K in O_2_/Ar followed by reduction at 873 K in H_2_/Ar the Ni–ZrO_2_ catalyst was exposed to CH_4_:CO_2_:Ar = 10/10/80 with a total flow rate of 25 ml min^−1^ at 873 K for 24 h (total pressure 1 bar). Figure 3 highlights the analysis obtained by gas chromatographic (GC) flame ionization detection (FID) and thermal conductivity (TCD) detection of reactants and products during methane dry reforming. It was observed that the CH_4_ and CO_2_ conversion as well as the production rate of H_2_ and CO hardly changed over time. The H_2_:CO ratio achieved was below one due to reverse water gas shift (RWGS) as a side reaction ($CO_{2} + H_{2} {\, \leftrightarrows\, } CO\, + \,H_{2} O;\quad \Delta H_{298 K}^{^\circ } = + 41 kJ/mol$). The C-balance (indicating coke formation when being below one) increased to nearly one during the first 3 h.

The turn-over-frequency (TOF) for H_2_ production based on the metallic Ni surface area after reduction at 873 K, normalized to the number of surface nickel atoms assuming 1.59 × 10^19^ nickel atoms/m^2^ [74], was initially 1.2 s^−1^, 1.2 s^−1^ after 3 h time on stream and 1.0 s^−1^ after 24 h time on stream.

**Fig. 4:**
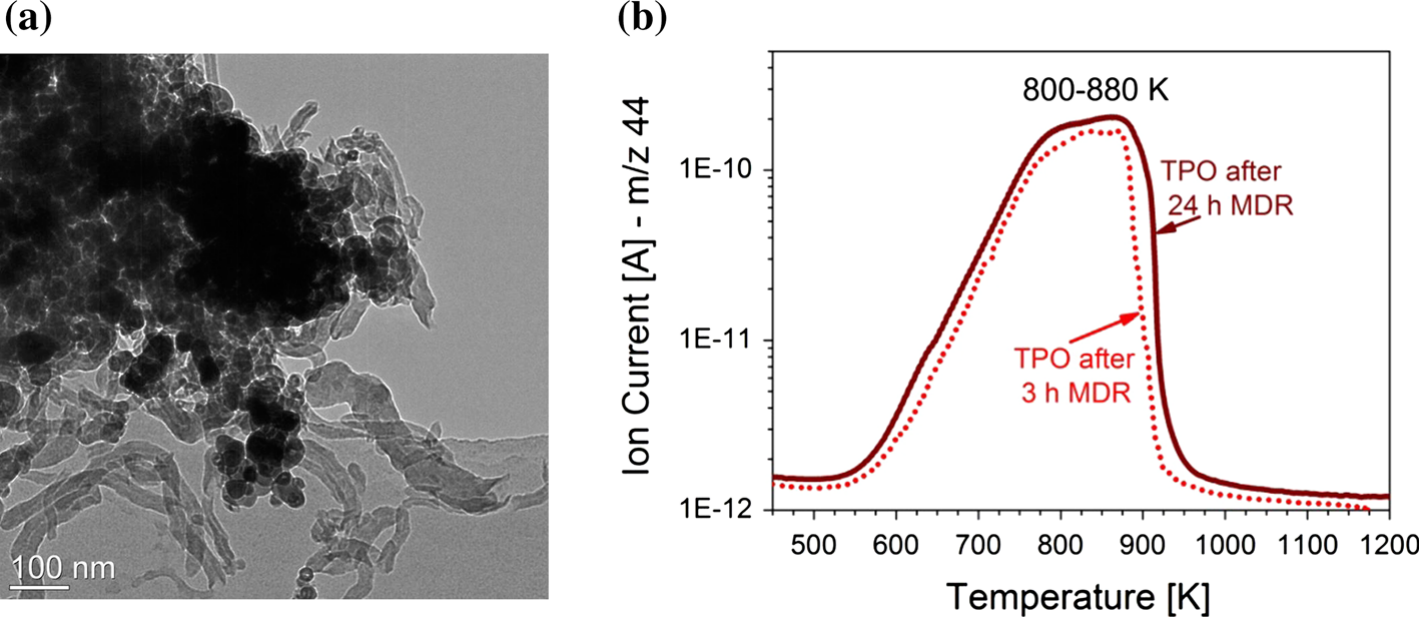
**a** TEM image of coked Ni–ZrO_2_ and **b** temperature programmed oxidation with 20 % O_2_ in Ar after exposure to CH_4_ and CO_2_ at 873 K

Figure 4a shows a TEM image of Ni–ZrO_2_ taken after dry reforming at 873 K (24 h). Filamentous carbon has formed and some Ni particles are located on top of the carbon nanofibers, tubes or rods.

After 24 h reaction in methane and carbon dioxide, temperature programmed oxidation (TPO) was performed with a heating rate of 5 K min^−1^ in 20 % O_2_ in Ar. TPO provides information about the amount of coke formed during the reaction and the temperature which is required to burn off the carbon species. This temperature is characteristic for the bond strength of carbon to the catalyst’s surface and for the nature of the carbon species. Figure 4b shows the CO_2_ production during TPO after dry reforming. Temperatures of about 800–880 K are needed to oxidize most of the coke. According to the literature, the first CO_2_ evolution maximum around 800–810 K can be assigned to graphitic carbon [75]. The CO_2_ evolution around 870–880 K can be assigned to the oxidation of whisker-type carbon which does not deactivate the nickel surface but rather causes a breakdown of the catalyst by pore plugging [76]. It is also interesting to note that the TPO acquired after a reaction time of 3 h is nearly identical indicating that coke deposition occurs rather rapid. Apparently, this type of coking does not deactivate the Ni surface but rather grows whiskers. The major problem of this catalyst is thus not the drop in catalytic activity but rather the physical blocking of the tube reactor. This was indeed observed when increasing the amount of catalyst in other catalytic tests.

**Fig. 5:**
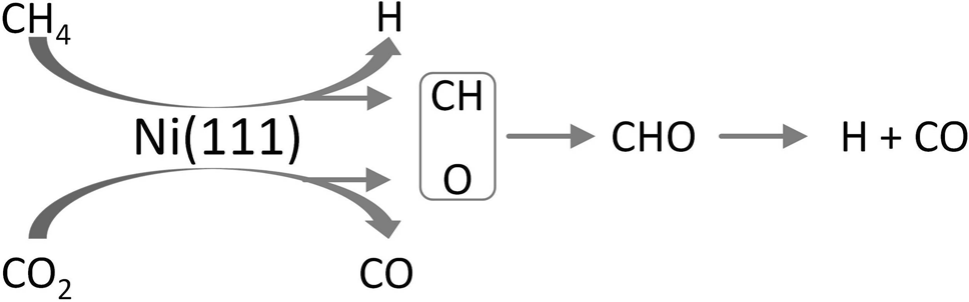
Simplified mechanism of CO_2_ reforming on Ni(111). Adapted from Wang et al. [35]

The activation of CH_4_ and CO_2_ as well as the reaction of these molecules on transition metal surfaces have been studied in great detail [34–39]. Methane activation on transition metal surfaces is characterized by a high activation barrier, a low sticking coefficient and a high hydrogen kinetic isotope effect [77]. Wang et al. [35] investigated the reaction pathways for MDR on Ni(111) by density functional theory and suggested a simplified reaction mechanism. The rate determining step was found to be CH_4_ dissociative adsorption. CH_3_, which is produced by CH_4_ dissociation prefers to dehydrogenate into CH_2_ which prefers to dehydrogenate to CH. However, CH prefers to be oxygenated to CHO by surface oxygen, which is formed by CO_2_ dissociation (without surface oxygen, dehydrogenation to C and H occurs). Dehydrogenation of CHO into H and CO then has a very low energy barrier on Ni(111). The mechanism proposed in this analysis of unsupported Ni is depicted in Fig. 5.

On supported catalysts, it is expected that the step of methane decomposition occurs on the metal particles [38, 39] while CO_2_ activation likely occurs on the support [36, 37, 78, 79]. Thus, the reaction between CH_x_ and “activated” CO_2_ might take place on the metal-support interface. For MDR over Pt/ZrO_2_/Al_2_O_3_ catalysts, a kinetic model based on this dual mechanism was successfully correlated with experimental data [7].

For CO_2_ activation, support materials with basic OH species increase the interaction of CO_2_ with the support and, therefore, increase the CO_2_ concentration on the surface, e.g. in form of carbonates, and also increase CO_2_ affinity for surface carbon which minimizes carbon accumulation ($CO_{2} + C_{{}} \,\leftrightarrows\, 2 CO\quad \Delta H_{298 K}^{^\circ } = + 171 kJ/mol$). [80].

Altogether, the reaction network is quite complex, and reforming, RWGS and Boudouard may occur simultaneously. Their relative contribution will strongly depend on the exact reaction conditions which also affect the state of the metal (CH_x_ coverage), of the support oxide (adsorbed carbonates, formates and OH), and of the metal-oxide interface.

#### Operando Spectroscopy During Methane Steam Reforming (MSR)

**Fig. 6:**
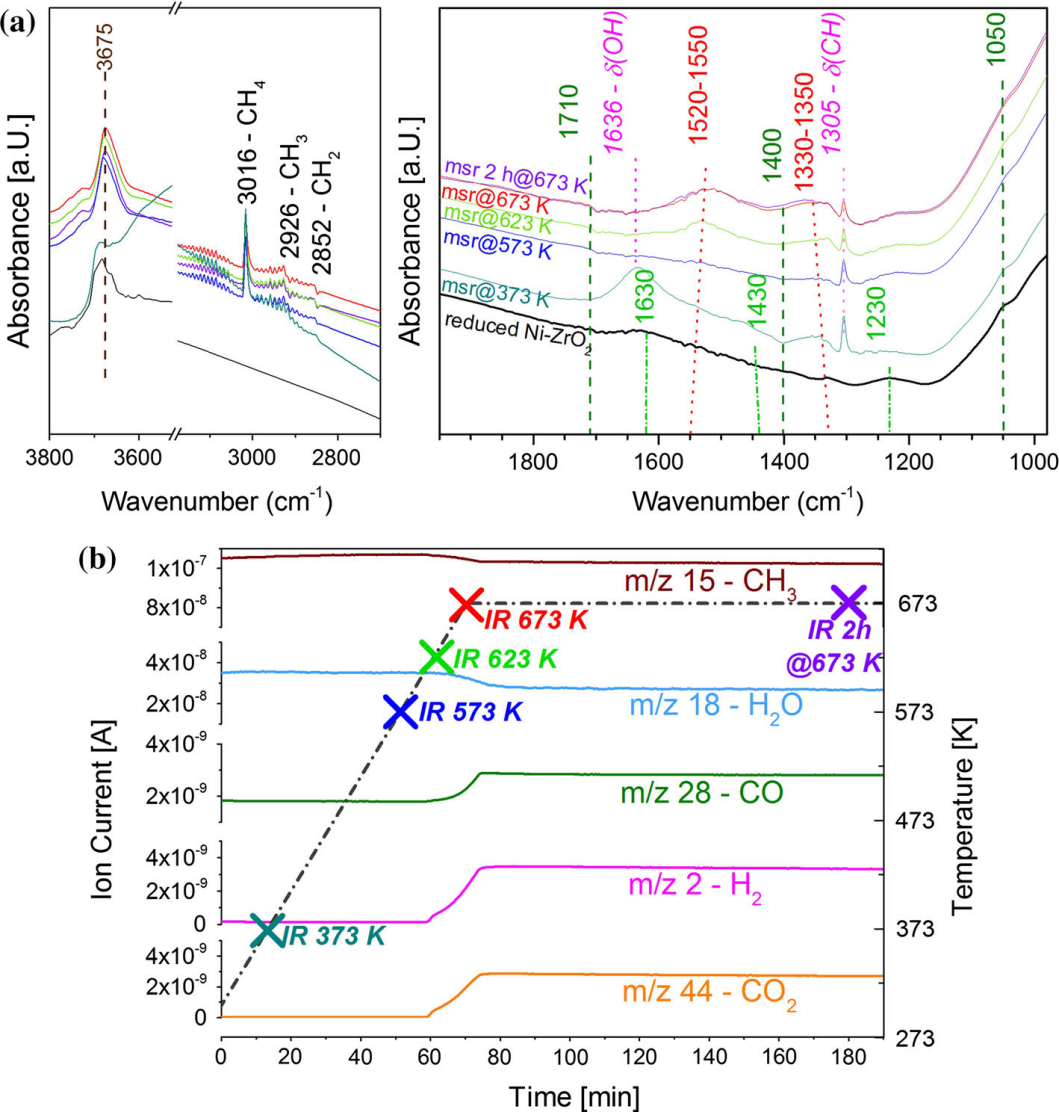
**a** Operando FTIR during methane steam reforming (MSR) reaction on Ni–ZrO_2_ at different temperatures. **b** Reactants/products followed by MS during temperature programmed MSR

The high reaction temperature of MDR (873 K) prevented to acquire operando FTIR spectra. In the following operando results are presented for methane steam reforming (MSR) on Ni–ZrO_2_,

$$CH_{4} + H_{2} O \to CO + 3 H_{2} \quad \Delta H_{298 K}^{^\circ } = + 206 kJ/mol,$$

obtained by simultaneous FTIR and MS measurements (Fig. 6). The pre-reduced catalyst was heated in a mixture of CH_4_ and H_2_O (~1.2 bar with a ratio of 1/3) from 300 to 673 K. As reference state, the FTIR spectrum of the reduced catalyst prior to the reaction is included.

The MSR reaction products are again CO and H_2_, i.e. both methane and water must be activated, e.g. via CH_4_ dehydrogenation (dissociative adsorption) and H_2_O dissociation. Once more, water gas shift (WGS; $CO_{{}} + H_{2} {\text{O }} \leftrightarrows CO_{2} + H_{2} \quad \Delta H_{298 K}^{^\circ } = - 41 kJ/mol$) may occur, accounting for the CO_2_ byproduct [38, 39].

On the reduced catalyst a small amount of bridged bidentate carbonates (at 1630, 1430 and 1230 cm^−1^) as well as bridged OH-groups (below 3700 cm^−1^) were observed by FTIR at room temperature (all typical of “residual” adsorbates on the ZrO_2_ support). In the reaction mixture at 373 K adsorbed water (broad peak around 1630 cm^−1^) but also adsorbed CH_3_ and CH_2_ groups were detected (i.e. methane decomposition starts already at low temperature), in addition to OH-groups. By further increasing the temperature to 573 K adsorbed water vanished from the surface, while the CH_x_ species remained. At about 623 K, H_2_, CO and CO_2_ formation (i.e. MSR and WGS) set in (detected by MS) and a new type of bidentate bicarbonate appeared on the surface (~1530 cm^−1^). The formed CO_2_ thus accounts for the (re)appearance of the (bi)carbonates. When temperature and reactivity were increasing the vibrations of bidentate *bi*carbonates shifted from 1550 to 1520 and from 1330 to 1350 cm^−1^. During 2 h of reaction at 673 K neither the adsorbates nor the reactivity towards MSR were changing, i.e. no deactivation was observed.

In summary, FTIR detected activated CH_4_ (CH_3_ and CH_2_), activated water (OH), as well as different bidentate (bi)carbonate species. The latter certainly originate from the water gas shift side reaction. Indeed, we have previously examined the reaction of the product CO with surface OH groups on an oxide surface, forming surface (bi)carbonates [79]. However, it remains unclear whether the bidentate (bi)carbonates are intermediates in the formation of CO [36, 37] or rather spectator species originating from readsorption of CO_2_. Additional concentration modulation experiments [81] would be required for a conclusive answer.

### UHV-Grown Model Catalysts of Ni–ZrO_2_/Pd_3_Zr(0001)

#### Preparation and Characterization

As described in the experimental section, the ZrO_2_ model support was prepared by oxidation/annealing of an adequate intermetallic compound, either Pd_3_Zr(0001) or Pt_3_Zr(0001), producing an ultrathin ZrO_2_ (O–Zr–O) trilayer. Using an electron beam evaporator, Ni nanoparticles were then grown on top of the ultrathin ZrO_2_ layer. The amount of deposited Ni (nominal thickness 3 Å) was controlled by a calibrated quartz microbalance. For further details concerning ZrO_2_ film preparation and cluster growth we refer to references [65, 66, 82, 83].

**Fig. 7:**
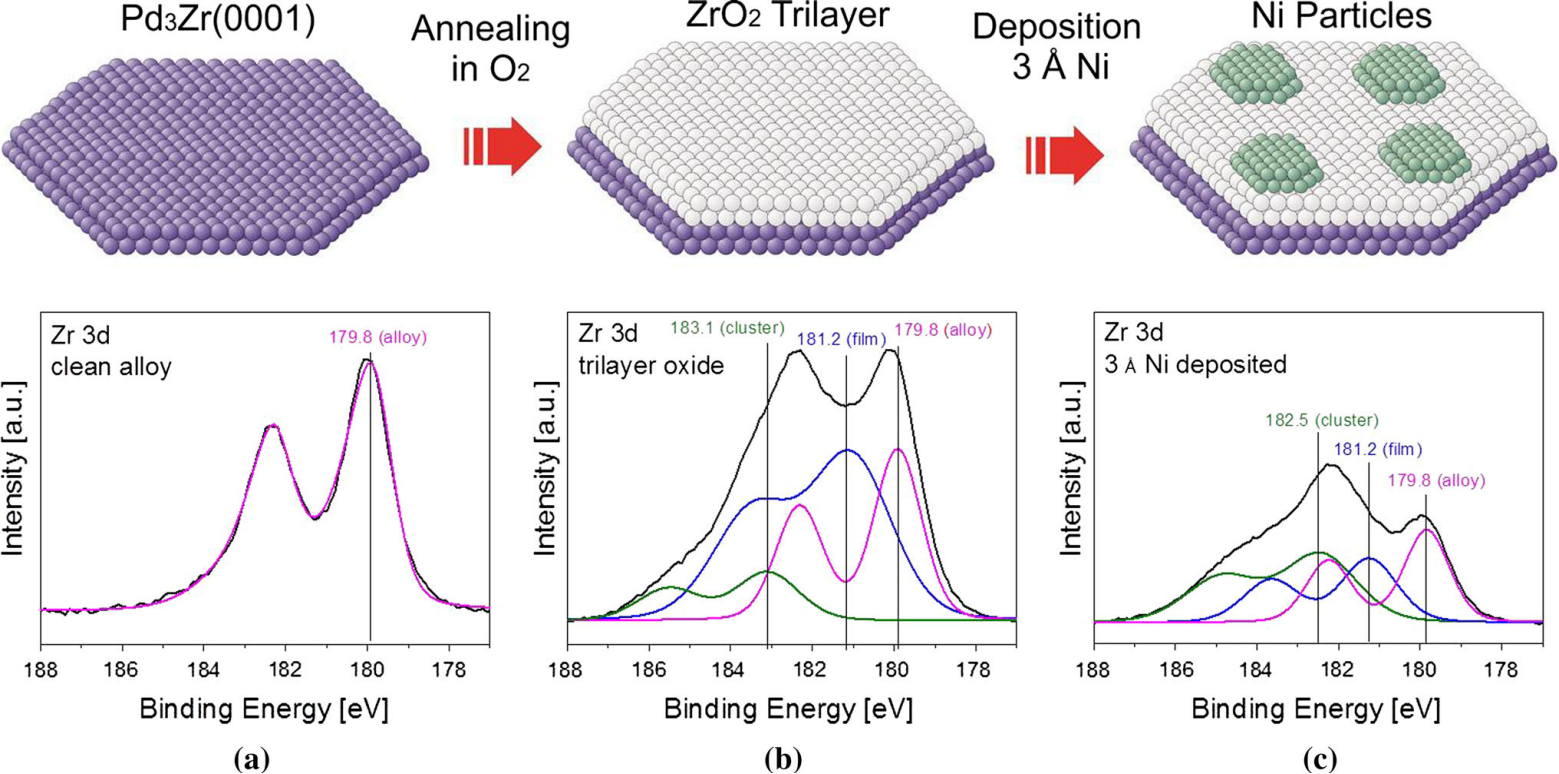
Preparation of the Ni–ZrO_2_/Pd_3_Zr(0001) model catalyst followed by XPS. *Top*: schematic illustration. *Bottom*: (**a**) XPS Zr3d signal of the clean alloy, and of the oxide before (**b**) and after Ni deposition (**c**). All spectra were fitted to distinguish between the respective components

The growth of the current ZrO_2_ thin film was monitored by XPS. Figure 7 shows Zr 3d spectra of the clean Pd_3_Zr alloy (left), of the ultrathin (O–Zr–O trilayer) ZrO_2_ (middle; after oxidation in 1 × 10^−7^ mbar of O_2_ at 673 K for 30 min, followed by annealing in UHV at 1073 K for 20 min), and after deposition of 3 Å Ni at 300 K onto the ZrO_2_ film (right).

The Pd_3_Zr alloy (left) is characterized by a peak at a BE of 179.8 eV, after oxidation there are additional components at 181.2 eV and 182.5/183.1 eV, due to the trilayer ZrO_2_ film and thicker ZrO_2_ clusters, respectively. Although oxide clusters are the minority species (about 10 % surface coverage), due to their thickness of several nanometers their contribution to the spectra is comparably larger (photoelectrons originate from multiple ZrO_2_ layers instead of a single ZrO_2_ layer). Using a laboratory X-ray source the inelastic mean free path (IMFP) of the photoelectrons is about 2 nm (sampling depth ~6 nm), thus the spectrum is still dominated by the alloy substrate. For more surface sensitive (synchrotron-based) XP spectra we refer to our study of the related ultrathin zirconia film on Pt_3_Zr [65]. The major difference of the two films is the higher surface defect density of ZrO_2_/Pd_3_Zr and, while for Pd_3_Zr the interlayer between the alloy and oxide is roughly stoichiometric, for Pt_3_Zr there is an interlayer of pure Pt.

**Fig. 8:**
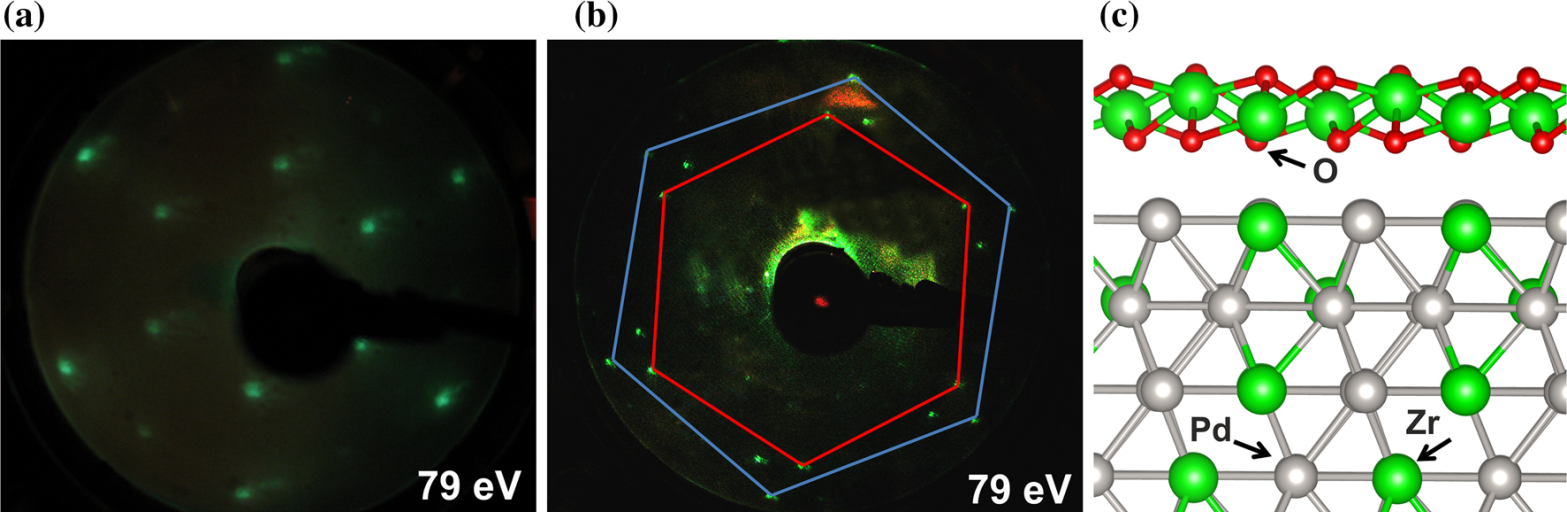
(**a**, **b**) LEED pattern and (**c**) schematics of the ultrathin ZrO_2_ (111) film on Pd_3_Zr (0001) annealed at 1073 K [LEED: oxide lattice (*red*); alloy substrate (*blue*)]

Figure 8a,b shows the LEED pattern (corresponding to Fig. 7a,b) measured with an electron energy of 79 eV, revealing the well-ordered (epitaxial) structure of the ultrathin oxide on the (0001) Pd_3_Zr surface. It displays the hexagonal reciprocal lattice of the oxide film (red hexagon) and of the alloy substrate (blue hexagon), with a ratio of the reciprocal lattices of oxide to substrate a_ox_/a_met_ of 1.556. According to scanning tunneling microscopy (STM) this corresponds to real space lattice parameters of 0.56 and 0.351 nm for oxide and alloy, respectively [66]. Furthermore, there are two oxide hexagonal lattices, rotated by an angle of 6.7° which correlates perfectly with the Fourier transform of STM images of the oxide film, indicating two rotational domains [65]. A structure model of the ZrO_2_ film on Pd_3_Zr, derived from combined STM and by density functional theory (DFT) studies [66], is shown in Fig. 8c. Based on the structural information, the specific XPS binding energies of (alloy supported) ZrO_2_ trilayers and of thicker ZrO_2_ layers could be explained by density functional calculations [65]. These XPS binding energies provide a useful reference for alloy (metal) supported ultrathin ZrO_2_ as well as for more bulk-like ZrO_2_ clusters.

Deposition of 3 Å Ni at room temperature induces a significant change in the Zr 3d region (Fig. 7c, top). Based on corresponding STM studies [83] of Ni cluster growth on ZrO_2_/Pt_3_Zr we expect that most (>80 %) of the ZrO_2_ support is covered by Ni, either in the form of closely spaced nanoparticles or, in case of coalescence, by large (~10 nm) connected Ni islands (with a height of about 0.2 nm). Locally, the oxide support is still accessible by the gas phase, however. On ZrO_2_/Pd_3_Zr, the nucleation density (~4 × 10^13^ cm^−2^) is about twice as high, so that a (nearly) closed and thin (~0.1 nm) Ni film is obtained.

The fitted Zr 3d region of the Ni-covered ZrO_2_/Pd_3_Zr is shown in Fig. 7c. It is evident that the (relative) intensity of the ZrO_2_ trilayer film (and that of the alloy substrate) strongly decreased while the signal of the ZrO_2_ clusters was much less affected (note that the exact geometry between sample, X-ray source and analyzer may vary between measurements, so that relative rather than absolute intensities should be compared).

The pronounced differences in the oxide trilayer and oxide cluster signals after Ni deposition can be explained by the different interplay between Ni and the underlying ZrO_2_. When Ni grows on the ultrathin oxide it attenuates the photoelectrons escaping from the underlying trilayer of ZrO_2_ (and from the Pd_3_Zr alloy beneath), as well as modifying the electronic and geometric structure of the trilayer (presumably because of its strong binding both to Zr and O [83]). The ZrO_2_ clusters exhibit a more bulk-like electronic structure, and seem less influenced by deposited Ni. This may be due, apart from their size of ~10 nm, to a different growth mode of Ni on ZrO_2_ clusters (e.g. with a lower nucleation density leaving more open space between Ni particles). A clear answer cannot be given at this point.

Photoemission spectra of the Ni 2p region (cf. Fig. 10) show that nickel is deposited in its metallic form, i.e. with the 2p region showing the satellite structure at 858.2 eV characteristic of metallic nickel. Note that on the technological catalyst, even after reduction at 673 K, part of the Ni surface remained oxidic.

#### CO Adsorption and Dissociation

**Fig. 9:**
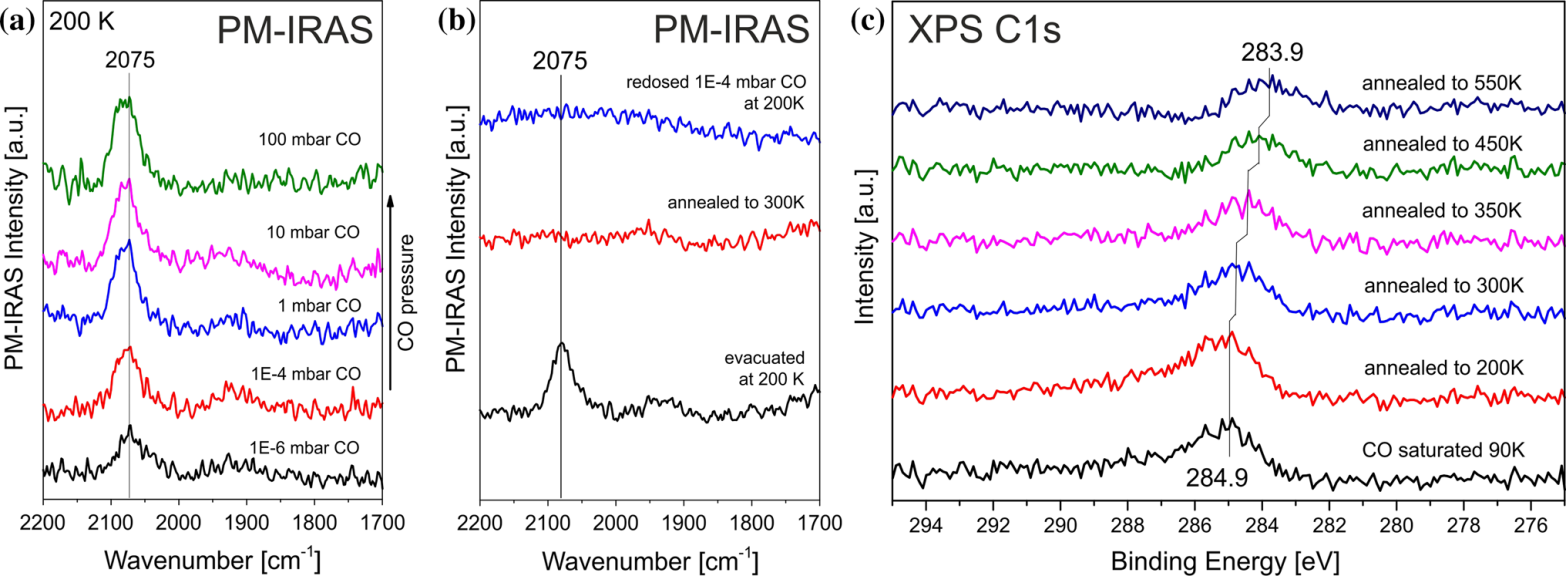
CO adsorption on the Ni–ZrO_2_/Pd_3_Zr(0001) model catalyst monitored by PM-IRAS and XPS. **a** CO adsorption at 200 K at increasing pressure; **b** PM-IRAS signal after adsorption and evacuation, followed by heating to 300 K. Upon readsorption at 200 K the CO band at 2075 cm^−1^ did not reappear; **c** C1 s spectra of stepwise annealing of the CO saturated surface followed by XPS

CO is, of course, a product of reforming and it is also involved in the (reverse) water gas shift reaction. In order to examine the interaction of the model reforming catalyst with gaseous CO, the freshly prepared 3 Å Ni/ZrO_2_/Pd_3_Zr sample was transferred to the high pressure cell [49] and cooled with liquid nitrogen to 200 K. Then, CO PM-IRAS spectra were acquired under isothermal conditions from 10^−6^ mbar to 100 mbar. As described in more detail in Ref. [50], PM-IRAS only displays the surface adsorbed species, whereas the gas phase signal is removed. The measured spectra (Fig. 9a) show a gradual increase of the absorption signal (or coverage of) CO at 2075 cm^−1^, which can be attributed to CO chemisorbed on top of individual nickel atoms. Signals of multiply-bonded CO (~1920 cm^−1^) were very broad and weak. CO adsorption on the ZrO_2_ trilayer (around 2190 cm^−1^ on powder ZrO_2_; cf. Fig. 2) was not observed, due to its desorption temperature of 155 K [65].

After evacuation of the reaction chamber and measuring another PM-IRAS spectrum at 200 K, on-top CO was still detected [Fig. 9b; which is expected since CO desorbs from Ni(111) and Ni(100) at ~425 K [64, 84]]. However, when the sample was gradually heated to higher temperatures in UHV, the signal of on-top CO vanished after heating to 300 K. Clearly, this cannot be due to CO desorption. Upon recooling and redosing 10^−4^ mbar (or higher) CO at 200 K the signal at 2075 cm^−1^ did not reappear, as shown in Fig. 9b. Possible explanations are either (1) that the Ni particles/islands had strongly sintered (less likely in view of the moderate temperatures) or (2) (more likely) that CO dissociated on nickel and that the formed coke covered the metal surface and hindered CO (re-)adsorption.

In order to examine the exact reason of the disappearance of the PM-IRAS absorption signal upon annealing/redosing, a fresh Ni/ZrO_2_/Pd_3_Zr model catalyst was prepared and the surface was saturated with CO at 90 K already in the preparation chamber (50 L CO exposure). Afterwards XP spectra of the C1 s and Ni2p regions were measured which revealed a peak at 284.9 eV characteristic of molecular CO (Fig. 9c), as well as a peak at 852.7 eV characteristic of metallic Ni (not shown). Afterwards, the model catalyst was successively heated in UHV up to 550 K. As shown in Fig. 9c a shift in C1 s binding energy towards lower binding energies could be observed, from 284.9 eV at 90 K (molecular CO) to 283.9 eV at 550 K (characteristic of sp^2^-bound carbon). This clearly shows that CO dissociates on nickel upon annealing, a well-known property, thereby forming a carbon over-layer on the nickel surface, which prevents further CO from adsorbing. During this experiment the Ni2p signal remained unaffected, i.e. there was no Ni sintering (which would reduce the intensity) and no formation of Ni carbide (which would induce a shift to 283.5 eV in C1 s [85]).

Previous studies of mbar pressure CO adsorption on supported Pd nanoparticles [52] and smooth and defect-rich Pd(111) single crystals [86, 87] have indicated the absence of CO dissociation on Pd. To confirm the hypothesis of coking on Ni, corresponding XPS experiments were thus performed for ZrO_2_ supported Pd nanoparticles. As expected, upon heating the Pd nanoparticles up to 550 K, CO desorption (~420 K) but no CO dissociation (carbon deposits) were detected by IRAS and XPS.

#### Ni Nanoparticle Stability

**Fig. 10:**
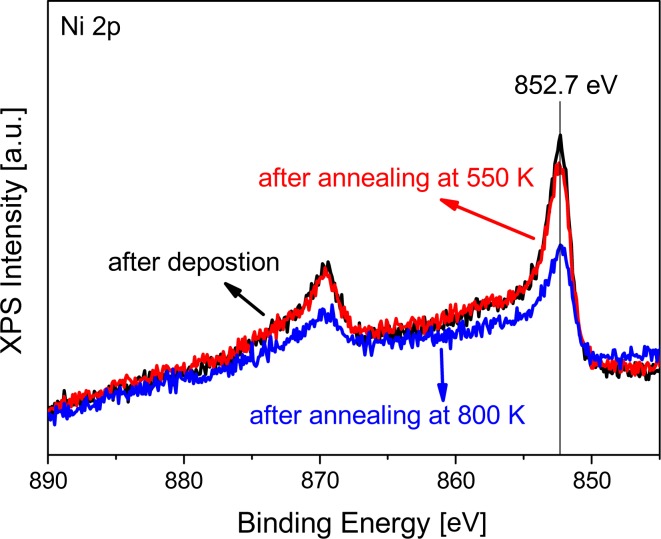
Ni 2p XP spectra of the pristine Ni–ZrO_2_/Pt_3_Zr(0001) model surface, and after annealing to 550 and 800 K in UHV

The thermal stability of Ni particles on ZrO_2_ was investigated using XPS (in this case for the Pt_3_Zr substrate, however). Figure 10 shows the corresponding Ni2p spectra, acquired after Ni deposition and after annealing in UHV to 550 K. Apparently, the Ni nanoparticles were stable up to 550 K, as indicated by the nearly identical spectra. However, annealing to 800 K induced a ~40 % decrease of the Ni 2p intensity, i.e. there was a strong “loss” of Ni. An apparent explanation would be a strong sintering of Ni nanoparticles. When the dispersion is reduced, the Ni2p signal decreases. However, it has been previously reported for ultrathin oxides that metal atoms may also diffuse through the ultrathin oxide and merge with the substrate, for example for Pd on alumina/NiAl [88], for Pd atoms on SiOx [89] and for Pd on thin FeO(111) film [90].

Indeed, corresponding STM studies of Ni clusters on ZrO_2_/Pt_3_Zr indicated that above 500 K metal atoms migrated through the oxide support into the alloy substrate [83]. We have thus modeled the XPS intensities of a 3 Å Ni layer above and below the ZrO_2_ support oxide. The calculations indicate that Ni migration to below the trilayer would indeed result in a ~40 % intensity decrease.

Once more, to confirm the hypothesis of “sub-oxide migration”, corresponding XPS experiments were performed for ZrO_2_ supported Pd nanoparticles (not shown). Upon heating the Pd nanoparticles to 500 K, the Pd 3d signal remained unaffected whereas annealing to 800 K again led to a ~32 % decrease of the XPS Pd 3d signal. Consequently, at high temperature the Pd atoms also migrated through the ultrathin oxide into the underlying alloy. Further experiments using low energy ion scattering (LEIS) or synchrotron XPS (which are both more surface sensitive than our laboratory XPS) are required to examine the “sub-oxide migration” in more detail.

## Discussion

We have examined Ni–ZrO_2_ reforming catalysts using both technological (powder) catalysts as well as model (single crystal based) catalysts [50, 57]. Both approaches have their specific benefits, the first providing results that may be technologically relevant and the latter providing access to examining specific processes on an atomic/molecular level. In both cases, similar characterization methods have been employed, e.g. microscopy/diffraction for structure analysis (HRTEM, XRD, LEED), X-ray photoelectron spectroscopy or fluorescence for composition analysis (XPS, EDX), and infrared spectroscopy and temperature-programmed methods for adsorbate characterization (FTIR, PM-IRAS, TPO).

The surface science approach to heterogeneous catalysis often provides very detailed insight into elementary processes. Typically, the catalytic systems are somewhat simplified, such as well-ordered (ultra)thin planar oxide films with deposited clean and well-shaped metal nanoparticles [even though these are complex systems when compared to (noble) metal single crystals]. Such model systems are not only accessible by many surface sensitive methods but can also be modelled straightforward by theoretical methods, which are typically indispensable to interpret and thoroughly explain the experimental data [60, 65, 91, 92]. Whether this approach is a realistic scenario for technological catalysis remains a matter of debate because often rather the disordered structures exhibit the highest activity.

Apart from the materials or complexity gap, in many cases model studies are performed in UHV and at low temperature, leading to the “pressure gap”. However, there are a number of “(near) atmospheric pressure surface science methods” available that enable to study functioning catalysts or, at least, model catalysts at elevated gas pressure and elevated temperature. Among the most frequently applied are PM-IRAS and SFG (sum frequency generation), both working up to 1 bar, and NAP-XPS (up to a few mbar) [50]. Nevertheless, operando spectroscopy is demanding in general, even for technological catalysts, and it is even more so for the low surface area model systems.

In the current case of Ni–ZrO_2_ reforming catalysts, the Ni particles in the powder catalyst are rather large (~20 nm) and also the Ni islands on the model ZrO_2_ cover most of the surface. Clearly, the “real” ZrO_2_ is hydroxylated whereas the model oxide is not. Nevertheless, routines have been developed to create OH groups on UHV grown (ultra)thin oxides as well.

With respect to CO adsorption at mbar pressure, differences were observed. CO adsorption on the technological Ni–ZrO_2_ was reversible, i.e. CO could be adsorbed, desorbed at higher temperature, and readsorbed without significant loss of adsorption capacity. There were also no indications of CO dissociation. In contrast, the Ni nanoparticles grown in UHV dissociated adsorbed CO already around room temperature, which led to coking and irreversible CO adsorption, thus readsorbing CO on the poisoned surface was not possible. Taking into account the well-known fact that CO dissociation preferentially occurs on Ni edges/steps this difference may be explained by the partial oxidation of the technological Ni nanoparticles (even after reduction Ni-oxide species were detected in addition to metallic Ni). Thus, it is likely that the edges of technological Ni particles are oxidized which prevents CO dissociation. Unfortunately, CO-FTIR was not able to differentiate step and terrace sites and also HRTEM was unable to demonstrate this (due to sample air exposure prior to microscopy).

Under methane dry reforming conditions, when hydrogen is present at high temperature, the Ni particles (and their edges) are fully reduced and may also partly dissociate the reforming product CO. Clearly, CH_4_ is the major carbon source and graphitic and whisker-type carbon were observed. Reverse water gas shift occurred in parallel and decreased the H_2_/CO ratio. For methane steam reforming operando (FTIR and MS) studies were performed, indicating activated CH_4_ (CH_3_ and CH_2_), activated water (OH), as well as different bidentate (bi)carbonate species. The latter originate from CO_2_ resulting from the water gas shift side reaction but it is unclear whether these are intermediates or spectator species.

Ni–ZrO_2_ model catalysts, prepared by surface science routes, turned out to be significantly more active for CO dissociation than their technological counterparts. When CO was adsorbed at pressures up to 100 mbar, both PM-IRAS and XPS revealed CO dissociation around room temperature and blocking of the Ni surface by carbon. This is most likely due to the fully metallic Ni particles whereas the technological particles were partially oxidized even after reduction at 673 K. CO adsorbed on the powder ZrO_2_ at room temperature, but it did not on the trilayer ZrO_2_ (CO desorption temperature of 155 K [65]).

A major limitation of most model systems is their thermal stability, however. For Ni–ZrO_2_/Pd_3_Zr the upper limit of stability is about 550 K. Around this temperature Ni atom migration through the ultrathin oxide (in)to the alloy substrate takes place and Ni is lost from the model catalyst surface. Such an effect does not occur for the technological catalyst and care must therefore be taken for model studies at higher temperature. Clearly, methane reforming cannot be studied in situ using the current model system.

## Conclusions

We have examined Ni–ZrO_2_ reforming catalysts, employing both technological materials (powders) as well as single crystal based surface science model catalysts. In both cases the Ni nanoparticles were rather large (20 nm or larger) but exhibited different properties with respect to CO dissociation. Whereas the partially oxidized “technological” Ni nanoparticles did not dissociate CO around room temperature, the fully metallic “surface science” Ni particles did, with the carbon deposits blocking further CO adsorption. For the technological samples methane dry and steam reforming were performed, with both reactions affected by (reverse) water gas shift, modifying the H_2_/CO ratio and producing CO_2_, respectively. For dry reforming whisker-type carbon was produced, operando studies of steam reforming revealed CH_x_, OH, and different bidentate (bi)carbonate species. The limited thermal stability of model Ni–ZrO_2_ prevented in situ reforming studies. Nevertheless, the XPS binding energies of alloy supported ultrathin trilayer (O–Zr–O) ZrO_2_ as well as those of more bulk-like ZrO_2_ clusters have been determined which provide useful reference data.

Altogether the combined technological and surface science approach is rewarding and it is fascinating to observe marked differences despite nominally the same Ni–ZrO_2_ composition. Of course, “Ni–ZrO_2_” is a very crude description missing important features like metal nanoparticle size and morphology, type of surface facets/steps, oxidation state, supporting oxide (surface) structure, hydroxylation, defect density, nature of metal-oxide interface sites, etc. Answering all these questions would provide the molecular details of how catalytic processes work and is thus a continuing mission.
